# Ultrahigh and kinetic-favorable adsorption for recycling urea using corncob-derived porous biochar

**DOI:** 10.1038/s41598-024-58538-1

**Published:** 2024-04-07

**Authors:** Xing Wang, Zhimin Chen, Chengqian Wang, Long Zhang

**Affiliations:** 1 Jilin Provincial Engineering Laboratory for the Complex Utilization of Petro-Resources and Biomass, School of Chemical Engineering, Changchun University of Technology, Changchun, 130012 People’s Republic of China; 2 Jilin Institute of Chemical Technology, Jilin, 132022 Jilin People’s Republic of China

**Keywords:** Corncob-derived porous biochar, Carbonization, Urea, Adsorption isotherm, Adsorption kinetics, Porous materials, Environmental chemistry

## Abstract

Insufficient attention has been given to the recycling of excess urea despite its potential detrimental effects on soil nutrient equilibrium, geological structure, and crop health. In this study, corncob-derived porous biochar (CPB), which is rich in surface functional groups, was prepared from biomass corncob in two steps as an adsorbent to remove urea from wastewater. Compared with the typical carbonization and activation processes, this process resulted in a higher yield of CPB and an ultrahigh adsorption capacity for urea. Response surface analysis was utilized to determine the optimal carbonization conditions, which were found to be 500 °C for 6 h with a heating rate of 15 °C/min. The exceptional adsorption capability of CPB can be ascribed to its porous structure and significant presence of oxygen-containing functional groups, which facilitate a synergistic interaction of physisorption and chemisorption. This adsorption phenomenon aligns with the Harkins–Jura isotherm model and adheres to pseudo-second order kinetics. CPB demonstrates potential as an adsorbent for the elimination of urea from wastewater in an economical and effective fashion.

## Introduction

Urea is used in fertilizers and widely employed in industrial chemical production. As a direct consequence of Given the demand for urea-derived products and the widespread consumption of urea in agriculture, the urea content in wastewater can reach 2 wt%^1,2^. Effluents containing urea induce eutrophication, leading to algal growth and a depletion of oxygen in rivers that is fatal to aquatic life^3^. In addition, excess urea in the soil can lead to increased nitrogen content, causing soil salinization and an imbalance in soil mineral nutrition, and also contribute to the formation of carcinogens that cause cancer^4^. Moreover, excess urea is converted to toxic ammonia and N_2_O, a Greenhouse Gas^5–7^. In the human body, urea is a product of protein metabolism and the main waste product accumulated during metabolic activity. Chronic kidney disease-induced kidney failure leads to elevated levels of urea that cannot be effectively metabolized or excreted by the body, necessitating the dependence of patients on hemodialysis. However, the retrieval of dialysis fluid poses a significant challenge in the process^8^.

Various methods have been devised thus far for the elimination of urea from waste streams, encompassing catalytic decomposition^9,10^, oxidative decomposition^11^, enzymatic decomposition^12^, and adsorption^13^. Taking an overview of these methodologies, adsorption is considered both effective and practical, involving a simple operating procedure, convenient working parameters, low energy requirements and insensitivity to toxic substances.

Biochar, a carbonaceous substance generated through the thermochemical transformation of biomass, is a versatile and recyclable material that has recently found many applications in various fields. Biochar possesses several distinctive features, including a substantial specific surface area, well-established pores, elevated cation exchange capacity, ample surface functional groups, and remarkable structural stability^14^. The advantages of biochar include its eco-friendly nature, reusability, multi-functional use and cost effectiveness^15,16^. However, the traditional method of template preparation is difficult to operate on a large scale due to the complexity of the preparation process and the high associated material costs, making it unfeasible to satisfy large-scale applications^17^.

Agricultural waste is a resource that is economical, renewable, and plentiful for biochar production^18,19^. This biomass consists mainly of hemicellulose, cellulose and lignin components. Numerous agricultural residues have been used to produce biochar, such as corncob^20^, waste wood^21^, rice straw^22^, and wheat straw^23^. Corncobs are the natural by-product of corn harvesting and production. At present, most of the corncob byproduct is used as fuel, and a smaller fraction is employed as raw material to produce furfural^24^. Xue-Lei Duan et al.^20^ employed ZnCl_2_ for chemical activation in order to produce corncob activated carbon possessing a substantial surface area. This activated carbon was subsequently utilized for the purpose of eliminating elemental mercury (HgO) from simulated flue gases. The achieved removal efficiency at a temperature of 150 °C was determined to be 91.4%.

In previous work, some of the common pyrochemical technologies used in biochar production have included torrefaction, gasification, flash carbonization, hydrothermal carbonization and pyrolysis^14,25^. The most widely used activators are metallic salts, acids^26–29^ and alkali compounds^30–32^. Yang et al.^33^ synthesized an activated carbon material by subjecting pistachio nuts to impregnation with ZnCl_2_ at a ratio of 1.5, followed by vacuum treatment at 500 °C for a duration of 2 h, resulting in a BET surface area of 2527 m^2^/g. Farnane et al.^34^ conducted a study on the activation of corncobs with H_3_PO_4_ for the purpose of removing methylene blue and malachite green dyes. The experimental findings demonstrated that the methylene blue exhibited a maximum monolayer adsorption capacity of 271.19 mg/g, while the malachite green displayed a higher capacity of 313.63 mg. While biochar has been used for urea removal, it has exhibited a low affinity for urea uptake. Kameda et al. employed a study wherein they utilized activated carbon spheres to facilitate the adsorptive separation of urea, creatinine, and uric acid, with the objective of recovering dialysis fluid. The researchers successfully achieved a maximum urea adsorption capacity of 1.63 mg/g^8^. Safwat reported the utilization of granular activated carbon and granular activated alumina with for the adsorption-based elimination of urea from wastewater, achieving a removal efficiency of 31% at a pH level of 9.0^35^.

In our prior research, it was discovered that the uncarbonized corncob-derived porous adsorbent (CPA) exhibits selective adsorption properties towards urea^36^, albeit with a lower adsorption capacity compared to other adsorbents. Building upon this previous work, the present study introduces a straightforward and effective approach for synthesizing porous biochar (CPB) using CPA as a precursor. The resulting CPB demonstrates an exceptionally high adsorption capacity for urea, with a yield reaching 34%. The CPB demonstrated a substantial specific surface area and was rich in oxygen-containing functional groups, resulting in a notable capacity for urea uptake (635.26 mg/g). The synergistic physical and chemical adsorption facilitated by the porous structure of CPB and the high surface concentration of oxygen-containing functional groups contributes to the enhancement of urea adsorption. The adsorption behavior of urea adhered to the Harkins–Jura isotherm and was described by a pseudo-second order kinetic model. The CPB developed in this study can be used for practical urea adsorption in wastewater treatment and hemodialysis recovery.

## Materials and methods

### Materials

The corncob (CC) used in this study was obtained from Jilin Xinbaoli Corncob Processing Plant (Jilin, China). Sodium hydroxide (NaOH)and hydrochloric acid (HCl) were all provided by Aladdin Reagent Co., Ltd. (AR, Shanghai, China). Urea standards and acetonitrile were purchased from Aladdin, (HPLC, Shanghai, China). Ammonium chloride (NH_4_Cl) and urea were purchased from Sinopharm Chemical Reagent Co, Ltd, (AR, Beijing, China).

### Preparation of the CPB

**Figure 1:**
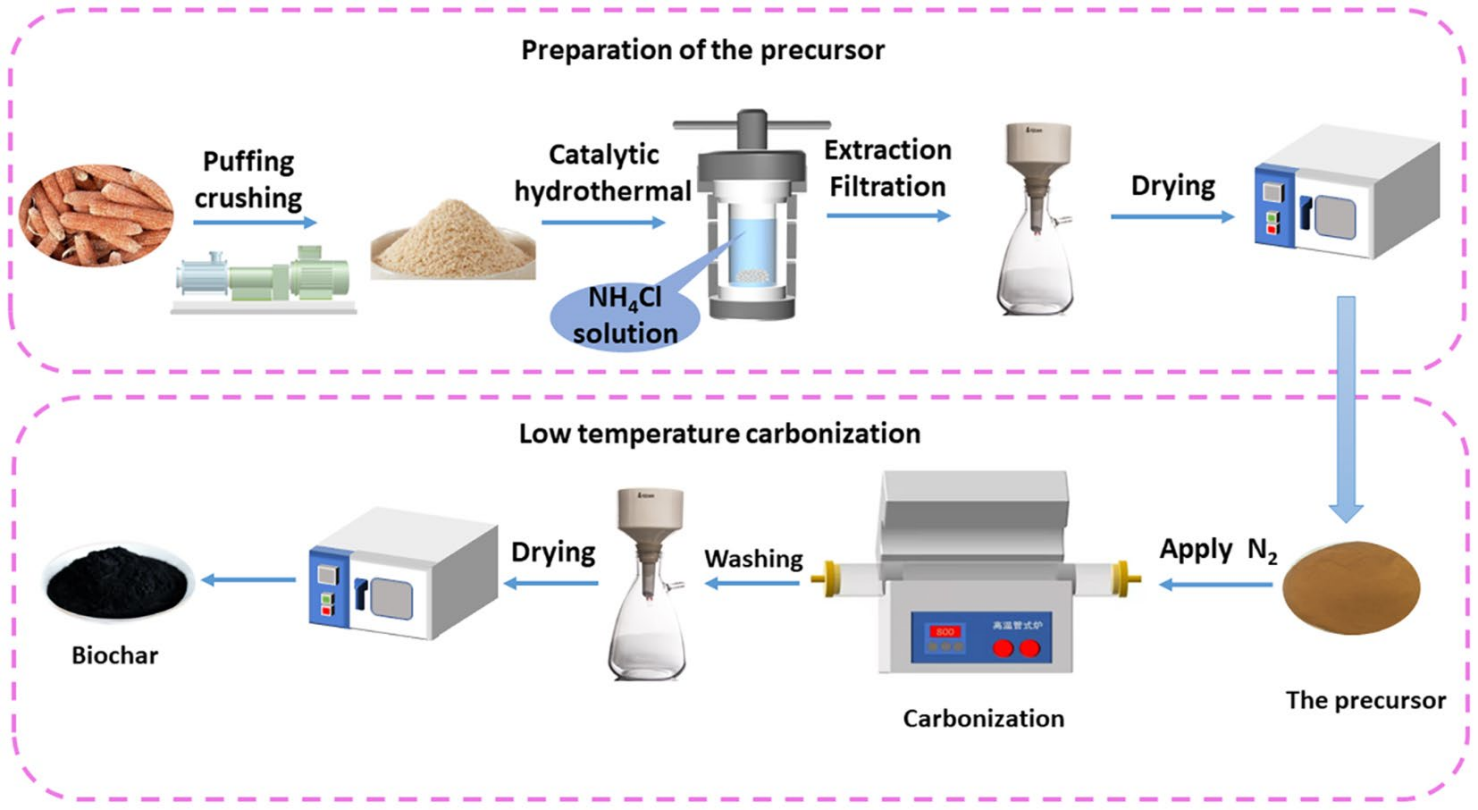
Preparation flow chart of the CPB.

In previous work^36^, the optimal process conditions for catalytic hydrothermal processes have been explored. A hydrothermal reactor with a capacity of 50 mL was utilized to conduct a reaction at a temperature of 142.6 °C for a duration of 2.6 h. The reaction involved the placement of a 2.0 g sample of CC powder (80–100 mesh) and 0.76 wt% of ammonium chloride catalyst. The solids were subjected to cooling at ambient temperature, followed by washing with deionized water and subsequent drying in an oven set at 80 °C, resulting in the acquisition of the corncob precursor (CPA). The CPA specimen was subjected to a controlled environment within a tube furnace (OTF-1200X, HF-Kejing, Hefei, China) while being exposed to a continuous flow of nitrogen gas. This exposure occurred for a specific duration (1–6 h), at a predetermined temperature(300–500 °C), and with a regulated heating rate(5–10 °C/min). The corncob porous biochar (CPB) was acquired through the process of solid washing with deionized water, followed by drying in an oven set at 80 °C. The CPB carbonized at 300, 400, 500 and 600 °C are denoted CPB03, CPB04, CPB05 and CPB06, respectively. The process is illustrated in Fig. 1. The dried CPB was weighed and used to calculate the yield of the biomass sorbent by Eq. 1.

1 $$ Yield = \frac{Mass \;of\; CPB}{{The\; initital\; mass\; of\; CC}}\; \times \;100\% $$

### Adsorption of urea

A conical flask with a capacity of 250 mL was utilized to contain a 100 mL sample of urea solution, which had a concentration of 150 mg/L. Subsequently, 50 mg of CPB was introduced into the flask after it was securely sealed with a stopper. The conical flask underwent oscillation within a constant temperature oscillator, wherein sample extraction occurred at specified time intervals, and the quantity of urea present in the solution was subsequently measured and calculated. To evaluate the urea adsorption capacity of CPB in an aqueous solution, an investigation was conducted to analyze the impacts of treatment duration, initial urea concentration, temperature and pH. The CPB was subjected to adsorption in a urea solution at a temperature of 25 °C and a concentration of 150 mg/L. Filtrates were collected at specific time intervals, 10, 20, 40, 60, 90, 120, 180, 360, and 720 min, to quantify the adsorption levels and ascertain the saturation capacity of urea Additionally, at concentrations of 50, 100, 150, 200, and 250 mg/L, after removal of the solution and saturation of the adsorption at 25 °C, the adsorption capacity was calculated. The assessment of uptake was performed at treatment temperatures of 20, 25, 30, and 35 °C, utilizing an initial urea concentration of 150 mg/L, throughout a time frame of adsorption saturation^36^. The effect of pH on the adsorption performance was investigated at 25 °C, 150 mg/L and saturated adsorption, and the pH was adjusted by using diluted solutions of 0.1 M HCl or 0.1 M NaOH^37^. All experiments were conducted in three sets of parallel experiments under identical conditions. The urea adsorption capacity was is calculated using Eq. 2,

2 $$ q = \frac{{\left( {C_{0} - C} \right)V}}{m} $$

the adsorption capacity of urea *q* (mg/g) is influenced by the concentration of urea at a specific time *C* (mg/mL), the initial urea concentration *C*_0_ (mg/mL), the volume of the urea solution *V* (mL), and the mass of the adsorbent *m* (g). Eq. 2 is utilized to derive the correlation between the equilibrium adsorption capacity (*q*_*e*_) and the equilibrium concentration (*C*_*e*_) of urea, which is subsequently presented as Eq. 3,

3 $$ q_{e} = \frac{{\left( {C_{0} - C_{e} } \right)V}}{m} $$

where the equilibrium urea adsorption capacity *q*_*e*_ (mg/g) is determined by considering various factors such as the volume of urea solution *V* (mL), the initial concentration of urea solution *C*_0_ (mg/L), the equilibrium adsorption *Ce* (mg/L), and the mass of absorbent *m* (g).

### Actual adsorption experiments

To examine the adsorption efficacy of CPB under authentic circumstances, adsorption experiments were conducted using both domestic wastewater and simulated human urine. The domestic wastewater sample was procured from Chaoyang District, Changchun City, Jilin Province, China, with a urea concentration of approximately 148.62 mg/L and a solution pH of 6.5. A volume of 100 mL of domestic wastewater was combined with 50 mg of CPB and allowed to reach saturation at a temperature of 25 °C, after which the adsorption quantity was determined. A solution was prepared by dissolving 231 mg urea in 100 mL of deionized water, resulting in a urea concentration of 2.31 mg/L at a pH of 7. This concentration is representative of the average urea concentration observed in the majority of patients with renal failure^38^. Additionally, a simulated urine solution consisting of 100 mL and 50 mg of CPB was subjected to adsorption at 37 °C until saturation, and the quantity of adsorbed material was determined.

### Characterization procedures

The microstructure of CC, CPA, and CPB samples, was assessed using a JSM-6510 (JEOL, Japan) scanning electron microscope (SEM). The observations were conducted under high vacuum conditions at 10 kV and 23 °C. Prior to analysis, the adsorbent underwent freeze-drying in a vacuum and was subsequently coated with a layer of gold. The nitrogen sorption measurements were conducted on a Micromeritics TriStar II 3020 analyzer to determine the specific surface area and pore size of the CPB. Prior to analysis, the samples were subjected to degassing and dehydration at a temperature of 120 °C for a duration of 12 h. The Brunauer–Emmett–Teller (BET) method was employed to calculate the Brunauer–Emmett–Teller surface area (SBET), while the Barret-Joyner-Halenda (BJH) method was utilized to determine the pore size distribution (PSD) of the CPB. To assess alterations in the functional groups pre- and post-carbonization, infrared spectroscopic analysis was conducted using a Nicolet IS10 (Thermo Fisher, USA) on CC, CPA and CPB samples following different carbonization temperatures, sample preparation involved the KBr compression method. A scan time of 64 was employed over the wavenumber range 500–4000 cm^−1^. The samples were kept under anhydrous conditions before analysis.

X-ray photoelectron spectroscopy (XPS) was employed to characterize the electron binding energy and element content of CPB and CPA (Thermofisher Escalab 250xi, USA). The specific parameters for the analysis are as follows, the total acquisition time is 2 min and 16 s, the number of scans conducted is 15, the source gun type used is A1 K Alpha, the spot size is 500 μm, the lens mode is set to standard, the analyzer mode is CAE, the pass energy is 30.0 eV, the energy step size is 0.1 eV, and the total number of energy steps taken is 181.

Zeta potential was determined by nanoparticle particle size and Zeta potential analyzer (Malvern Zetasizer Nano ZS90, UK). The samples that had undergone adsorption were suspended in ultrapure water, and the resultant suspension was transferred into a pristine zeta cell. Subsequently, measurements were conducted at a temperature of 25 °C for a duration of 70 s, with the identical sample being measured thrice.

The concentration of urea in the filtrate was analyzed using high-performance liquid chromatography (HPLC)^39,40^, with an Agilent Zorbax 250 × 4.6 mm id amine (NH_2_) column containing 5 µm particles. The mobile phase consisted of 95% acetonitrile and 5% water, flowing at a rate of 1.0 mL/min. The column temperature was maintained at 25 °C, and urea was detected using UV detection at 195 nm, resulting in an error of less than 1.0%.

## Results and discussion

### Micro structure and properties of CPB

**Figure 2:**
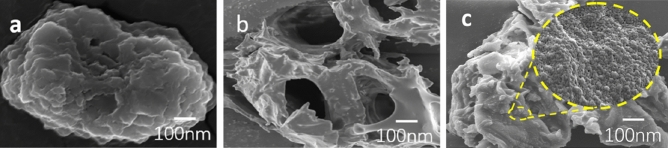
SEM images of (**a**) the CC; (**b**) the CPA; (**c**) and the CPB05 with optimal preparation parameters.

As shown in Fig. 2, it can be seen that the surface of untreated CC is lamellar and essentially without pores. Following catalytic hydrothermal treatment of CPA, the resulting pore structure becomes pronounced, with larger pores evident. In comparison, CPB exhibits smaller pore sizes, with a notable increase in pores measuring 100 nm or less, which are advantageous for urea adsorption.

**Figure 3:**
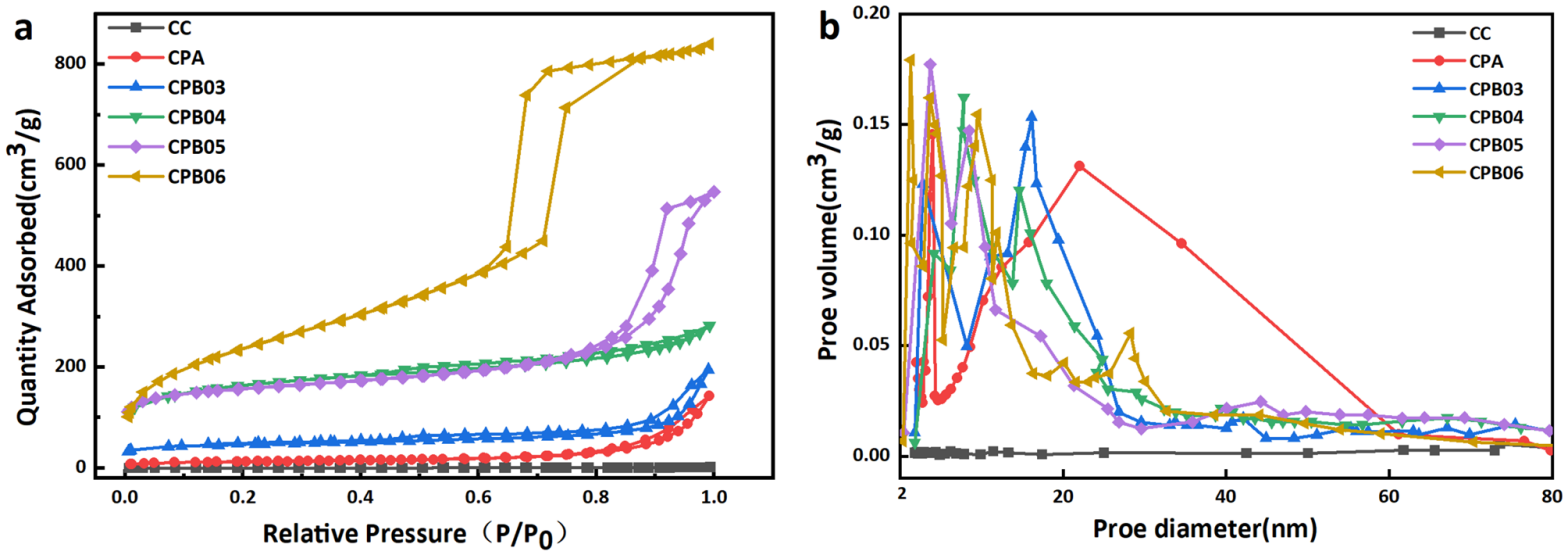
The CPB at different carbonization temperature (**a**) N_2_ adsorption–desorption isotherms and (**b**) pore size distribution curves of.

Fig. 3 displays the outcomes of the N_2_ adsorption–desorption measurement, elucidating the pore characteristics of the raw materials, precursors, and the CPB produced at varying carbonization temperatures. The analysis of Fig. 3a reveals that the initial CC resulted in a type III isotherm, which suggests a limited level of interaction between the adsorbent and adsorbate. The CPA demonstrated a type II isotherm accompanied by a H3 type hysteresis loop, suggesting either a non-porous nature or the existence of large pores. The isotherms for CPB03 correspond to type IV with a H3 hysteresis loop, suggesting a predominant mesoporous character, and CPB04 exhibited a type IV isotherm accompanied by a H4 type hysteresis loop, suggesting the coexistence of micropores and mesopores. Furthermore, CPB05 and CPB06 exhibited a type IV isotherm accompanied by a H1 type hysteresis loop, suggesting a narrower distribution of mesopores. It is known that stratified porous carbon materials with multiple pore sizes can significantly shorten the mass transfer path, increasing the mass transfer rate with a greater available surface area^41^. It is known that stratified porous carbon materials with multiple pore sizes can significantly shorten the mass transfer path, increasing the mass transfer rate with a greater available surface area The porosity characteristics of the different samples are further elucidated by the entries in Fig. 3b. It can be seen that CC shows no evident porosity and CPA predominantly exhibit macropores larger than 50 nm, while all CPBs are characterized by mesopores below 50 nm, which correspond well with the nitrogen adsorption curves. This distribution of pore size should facilitate the subsequent adsorption of urea. The pore structure parameters are listed in Table 1.

**Table 1:** The pore structure parameters.

Sample	S_BET_* (m^2^/g)	V_total_*(cm^3^/g)	D_ave_* (nm)
CC	1.80		
CPA	46.85	0.01	1.01
CPB03	156.96	0.05	1.39
CPB04	462.57	0.11	0.94
CPB05	533.67	0.11	3.70
CPB06	561.17	0.10	1.40

*S_BET_: BET surface area; V_total_.: Total pore volume; D_ave_: Average pore diameter.

In this study, a catalytic hydrothermal removal of most of the hemicellulose was achieved at 140 °C. From a consideration of Fig. 3b, it is evident that two peaks appear at 0–60 nm for CPA after hydrothermal treatment when compared with CC, indicating a significant increase in the mesopore component, but an uneven distribution of pore size can result in lower urea adsorption. The subsequent low temperature carbonization resulted in the removal of residual hemicellulose and cellulose at 200–450 °C, and a small amount of lignin was removed at 300–500 °C^42–44^. The specific surface area exhibited an upward trend as the temperature ranged from 300 to 500 °C, as depicted in Fig. 3b, with a narrower pore size distribution and a shift from mesopores to micropores. Sample CPB05 exhibited a large number of mesopores with a lesser micropore content, favoring multilayer adsorption^45^. With an increase in the carbonization temperature to 600 °C, there was a slight increase in the specific surface area, accompanied by a greater proportion of pore size (< 20 nm,) within the microporous region. In order to further verify the optimal carbonization temperature, urea adsorption was carried out on CPB06. The saturation adsorption capacity of CPB06 (566.32 mg/g) was lower than that of CPB05(635.26 mg/g). This implies that an elevated carbonization temperature has the potential to diminish the presence of oxygen-containing functional groups^46^, consequently leading to a decrease in urea uptake.

**Figure 4:**
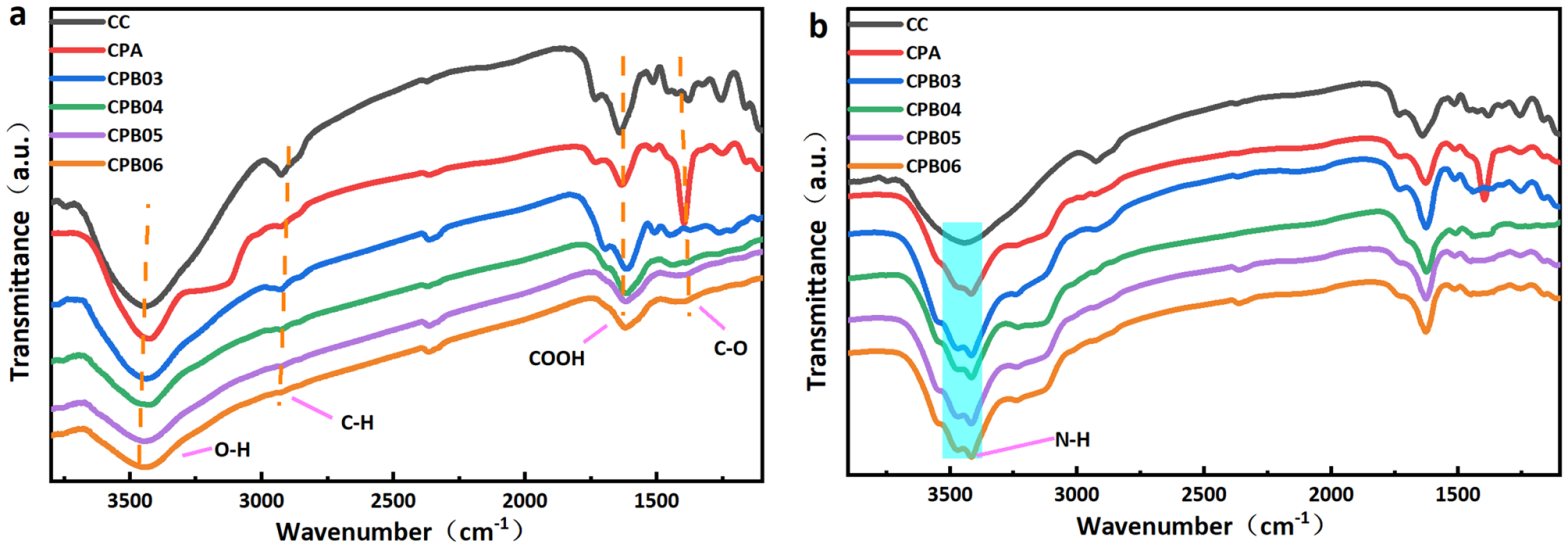
(**a**) FT-IR spectra of the CPB from different carbonization temperatures and (**b**) after urea absorption.

In order to confirm the existence of oxygen-containing functional groups, FT-IR analyses were conducted. Fig. 4 displays the FT-IR spectra of the CPB obtained at various carbonization temperatures. The spectral bands observed within the wavenumber range of 1636–1520 cm^−1^ can be ascribed to the stretching vibrations of carboxylic acid (COOH) functional groups, while the bands encompassing the wavenumber range of 1000–1450 cm^−1^ are indicative of bending vibrations associated with C–O bonds present in hydroxyl, ester, and ether functional groups, as well as O–H bonds^47^. The spectral bands observed at wavenumbers of 2922–2855 cm^−1^ and 3000–3700 cm^−1^ can be attributed to the vibrational modes of aliphatic C–H and O–H (hydroxyl or carboxyl) functional groups, respectively^48^. In the CPA stage, most of the functional groups are retained on the surface. When the carbonization temperature was increased from 300 to 600 °C, only the band at 1000–1450 cm^−1^ largely disappeared. This indicates that a large number of oxygen-containing functional groups (hydroxyl and carboxyl groups) are retained, which promote the efficient adsorption of urea by forming hydrogen bonds.

The presence of NH is indicated by a distinct peak at 3330 cm^−1^ in Fig. 4b. This serves to indicate that the carboxyl and amino groups on CPB have undergone dehydration, as presented in Eq. 4. Hydrogen bonding between the hydroxyl group on CPB and the amine group of urea caused a shift in the hydroxyl peak. Moreover, the observed displacement of the carbonyl peak can potentially be attributed to the formation of hydrogen bonds between the carbonyl group in CPB and the amine group in urea, thereby augmenting the adsorption capacity of urea.4

**Figure 5:**
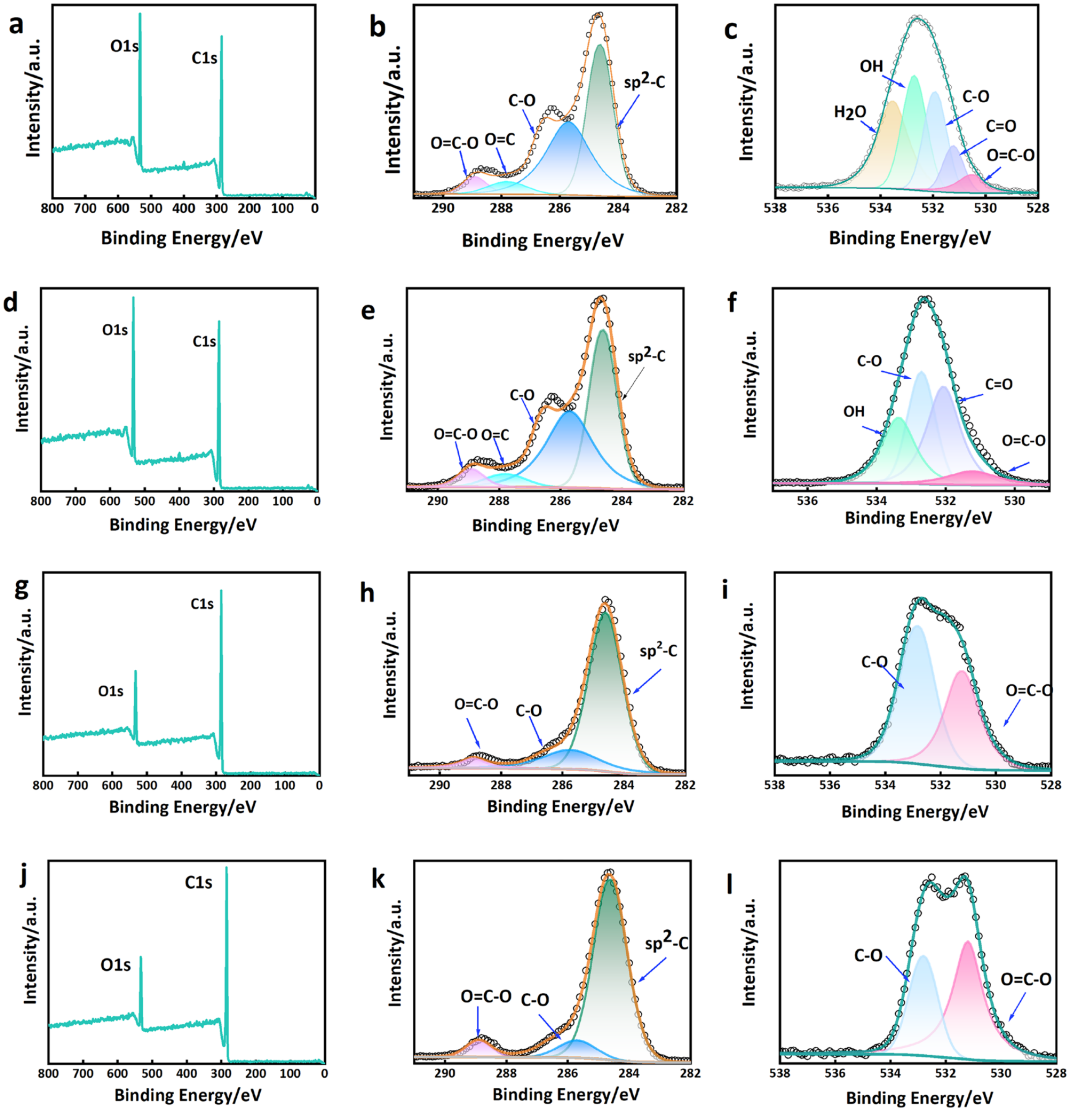
(**a**) XPS spectra, (**b**) C1*s* spectra, and (**c**) O1*s* spectra of CPA, (**d**) XPS spectra, (**e**) C1*s* spectra, and (**f**) O1*s* spectra of CPB03, (**g**) XPS spectra, (**h**) C1*s* spectra, and (**i**) O1*s* spectra of CPB04, (**j**) XPS spectra, (**k**) C1*s* spectra, and (**l**) O1*s* spectra of CPB05.

**Figure 6:**
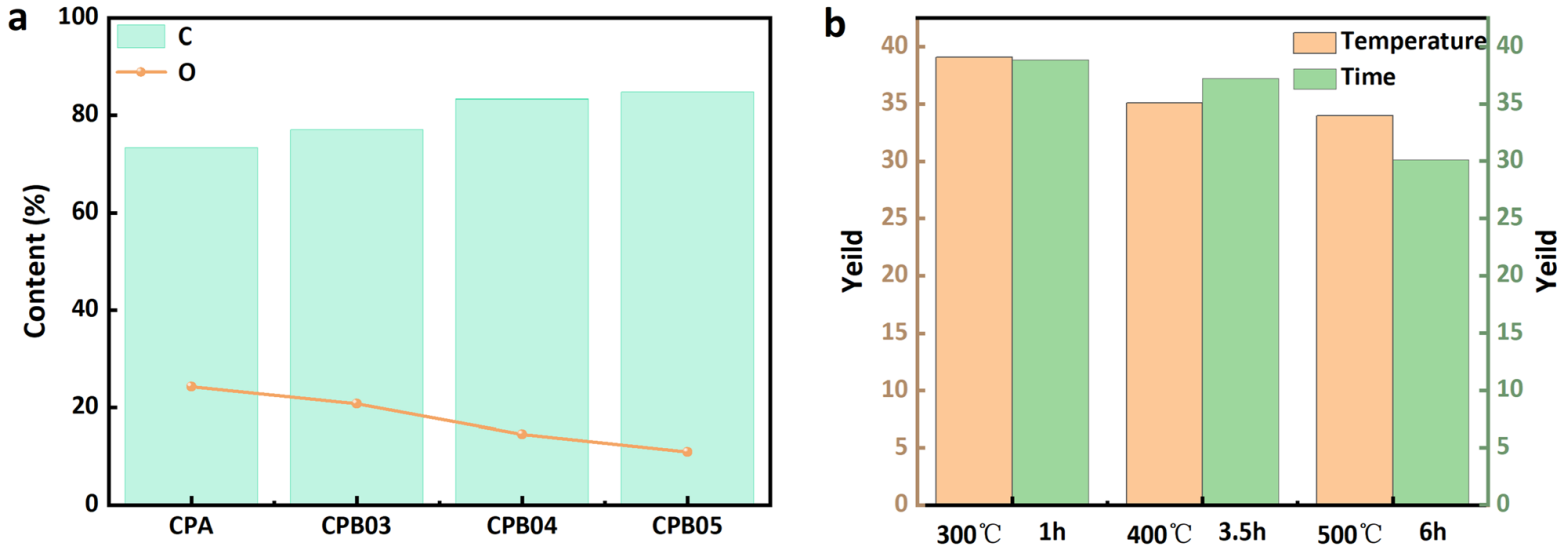
(**a**) Content of C and O in CP and CPB (at various temperatures carbonized 6 h), (**b**) carbonization product yield of CPB carbonized at various temperatures for 6 h and at various times for 500 °C.

The purpose of conducting XPS analysis was to investigate the surface properties of CPA and CPB, with the objective of offering supplementary evidence concerning the modifications in oxygen-containing functional groups. Fig. 5a,d,g,j exhibit conspicuous C1*s* and O1*s* peaks at 285 eV and 533 eV for both CPA and CPB, with the intensity of the O1s peaks diminishing as the carbonization temperature rises. The fitting outcomes of the C1*s* and O1*s* fine scanning spectra for CPA are presented in Fig. 5b,c, respectively^36^. The findings of the study revealed the presence of three distinct component peaks at 284.6 eV, 285.7 eV, and 288.9 eV in the C1s spectrum, indicating the presence of *sp*^2^-C, C–O, and O=C–O^49^. In contrast, the O1s spectrum exhibited five separate component peaks at 531.2 eV, 531.9 eV, 532.8 eV, 533.4 eV, and 535.5 eV, corresponding to O=C–O, C=O, C–O, OH, and H_2_O^45^. The corresponding fine scanning spectral fits of the C1s and O1s orbitals for CPB03 are depicted in Fig. 5e,f, respectively. The C1*s* peaks at 284.6 eV, 285.7 eV, 287.8 eV, and 288.9 eV exhibit four distinct component peaks, namely *sp*^2^-C, C–O, C=O, and O=C–O. Similarly, the O1s peaks at 531.2 eV, 531.9 eV, and 532.8 eV display four separate component peaks, specifically O=C–O, C=O, C–O, and OH. The C1s spectra of CPB04 and CPB05, as depicted in Fig. 5h,k, can be disassembled and reconstructed into three distinct component peaks, 284.6 eV (*sp*^2^-*C*), 285.7 eV (C–O), and 288.9 eV (O=C–O). Likewise, the O1s peaks in both Fig. 5i,l exhibit two distinct component peaks at 531.2 eV (O=C–O) and 532.8 eV (C–O). Fig. 5 provides visual evidence of the substantial presence of oxygen-containing functional groups in CPB, as indicated by the carbon and oxygen content displayed in Fig. 6a. It can be observed that the quantity of oxygen-containing functional groups decreases with increasing carbonization temperature. Notably, CPB05 retains an oxygen content of up to 10%, and these oxygen-containing functional groups play a crucial role in facilitating hydrogen bonding with urea and augmenting the adsorption capacity.

The carbonization yields at different carbonization temperatures and times calculated using Eq. 1 are shown in Fig. 6b. The yield decreased from 40 to 30% as the temperature and time were increased. In previous related studies on biochar, few researchers have explicitly quoted yields.

### Performance of CPB in urea adsorption

#### Effect of optimization the parameters on the uptake capacity of CPB

The carbonation parameters can impact on ultimate adsorption performance. In order to optimize carbonation conditions, urea adsorption was carried out at 25 °C with a urea concentration of 150 mg/L, 50 mg CPB and an adsorption time of 2 h. The results were then optimized by adjusting the carbonation temperature, time, and heating rate using the Box-Behnken design (BBD). The influence of the carbonization parameters on urea adsorption was determined by response surface method (RSM) analysis. The range and mean values of the independent variables according to BBD are listed in Table 2, and the experimental design and results are listed in Table 3; A, B, and C represent the carbonization temperature, carbonization time and heating rate, respectively. The proposed model by the experimental design for urea adsorption from the multiple regression analysis of the experimental data is shown in Eq. 5,

5 $$ \begin{aligned} {\text{Urea adsorption capacity}} & = {375}.{65} + {57}.0{7}A + {39}.{1}0B + {32}.{28}C \\ & \quad - {16}.{71}AB + {36}.{26}AC + {32}.{22}BC + {28}.{85}A^{{2}} + {4}0.{67}B^{{2}} - {1}.{5}0C^{{2}} \\ \end{aligned} $$

**Table 2:** Evaluation for optimizing carbonization parameters.

Variables	Levels		
	− 1	0	1
A: Carbonization temperature (°C)	300	400	500
B: Carbonization time (h)	1	3.5	6
C: Heating rate (°C/min)	5	10	15

**Table 3:** Experimental design for optimization of carbonization parameters.

Run	A: Carbonization temperature (°C)	B: Carbonization time (h)	C: Heating rate (℃/min)	Urea adsorption (mg/g)
1	300.00	1.00	10.00	288.83
2	300.00	3.50	15.00	376.50
3	300.00	3.50	5.00	359.54
4	300.00	6.00	10.00	443.18
5	400.00	6.00	5.00	380.50
6	400.00	3.50	10.00	375.65
7	400.00	3.50	10.00	375.65
8	400.00	1.00	15.00	384.70
9	400.00	3.50	10.00	375.65
10	400.00	3.50	10.00	375.65
11	400.00	3.50	10.00	375.65
12	400.00	1.00	5.00	409.49
13	400.00	6.00	15.00	484.60
14	500.00	6.00	10.00	568.09
15	500.00	1.00	10.00	480.59
16	500.00	3.50	5.00	356.98
17	500.00	3.50	15.00	518.98

The F-test and analysis of variance (ANOVA) were conducted to evaluate the suitability and credibility of the regression models. The outcomes of the ANOVA for urea sorption are displayed in Table 4. The *p*-value of the model was statistically analyzed to be less than 0.05, confirming the applicability and predictive significance of the model. The *R*^2^ value of 0.7423 for urea sorption was greater than the adjusted *R*^2^ value of 0.5877, the standard deviation in the response variable of urea adsorption is 44.55, indicating that the model was not over-fitted^50^. The order of influence of the three independent variables on urea sorption, i.e. A > B > C, is shown in Table 3.

**Table 4:** ANOVA results of RSM.

Source	Sum of Squares	D_f_*	Mean Square	F Value	p-value Probability > F	Significant*
Model	68,199.60	9	7577.73	6.03	0.0136	significant
A	26,059.82	1	26,059.82	20.73	0.0026	
B	12,228.27	1	12,228.27	9.73	0.0169	
C	8337.47	1	8337.47	6.63	0.0367	
AB	1117.16	1	1117.16	0.8888	0.3772	
AC	5258.55	1	5258.55	4.18	0.0801	
BC	4153.45	1	4153.45	3.30	0.1119	
A^2^	3504.78	1	3504.78	2.79	0.1389	
B^2^	6965.79	1	6965.79	5.45	0.0508	
C^2^	9.46	1	9.46	0.0075	0.9333	
Residual	8798.31	7	1256.90			
Lack of Fit	8798.31	3	2932.77			
Pure Error	0	4	0			
Cor Total	76,997.91	16				

*Df degree of freedom; Significant (*p* < 0.05).

**Figure 7:**
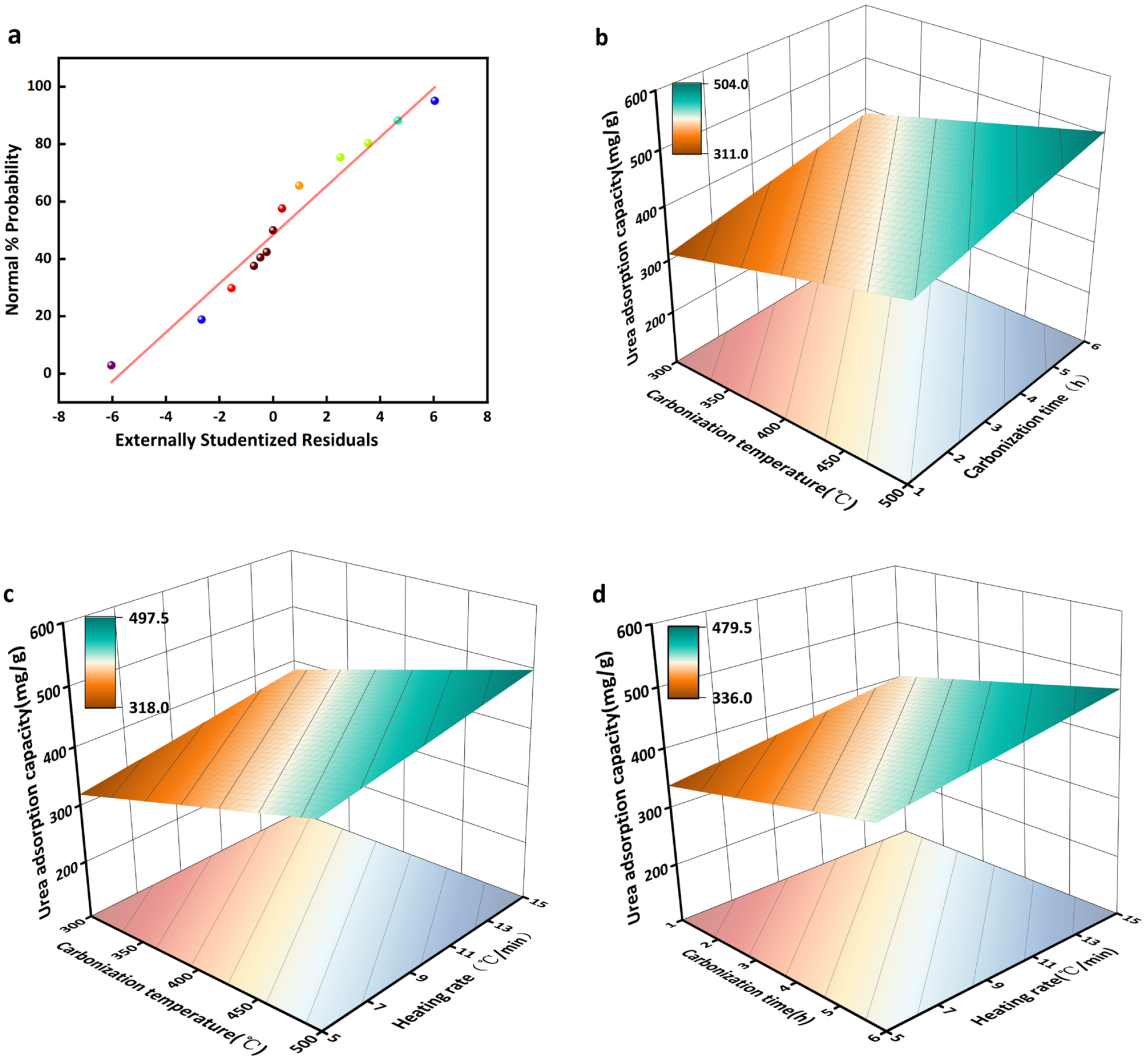
Normal probability (**a**) plot versus residua and 3D Response surface plots (**b**) response of carbonization temperature and carbonization time at constant heating rate (**c**) response of carbonization temperature and heating rate at constant carbonization time (**d**) response of carbonization time and heating rate at carbonization temperature.

Fig. 7a reveals a normal probability distribution, which establishes that the data points are evenly distributed above and below a straight line, indicating that the errors are normally distributed along this line. Hence, the model has predicted the response adequately. The response of various carbonation parameters to urea sorption is shown in Fig. 7. It can be seen that adsorption performance strongly depended on carbonation conditions. All three variables had a positive response to urea sorption. Increasing the carbonation temperature (Fig. 7b), time (Fig. 7c), and heating rate (Fig. 7d) can lead to improvements in urea adsorption. The respective values of the different variables have been optimized by RSM in order to maximize the adsorption of urea and the optimal values are as follows, carbonation temperature 500 °C, time 6 h, and heating rate 15 °C/min, with a urea adsorption capacity of 587.88 mg/g. The experimental adsorption capacity of the CPB achieved under the optimal carbonization conditions was 586.46 mg/g. The observed deviation of 0.24% between the obtained and anticipated outcomes serves as evidence of the precision achieved through the utilization of optimized parameters.

#### Effect of adsorption time on the uptake capacity of CPB

**Figure 8:**
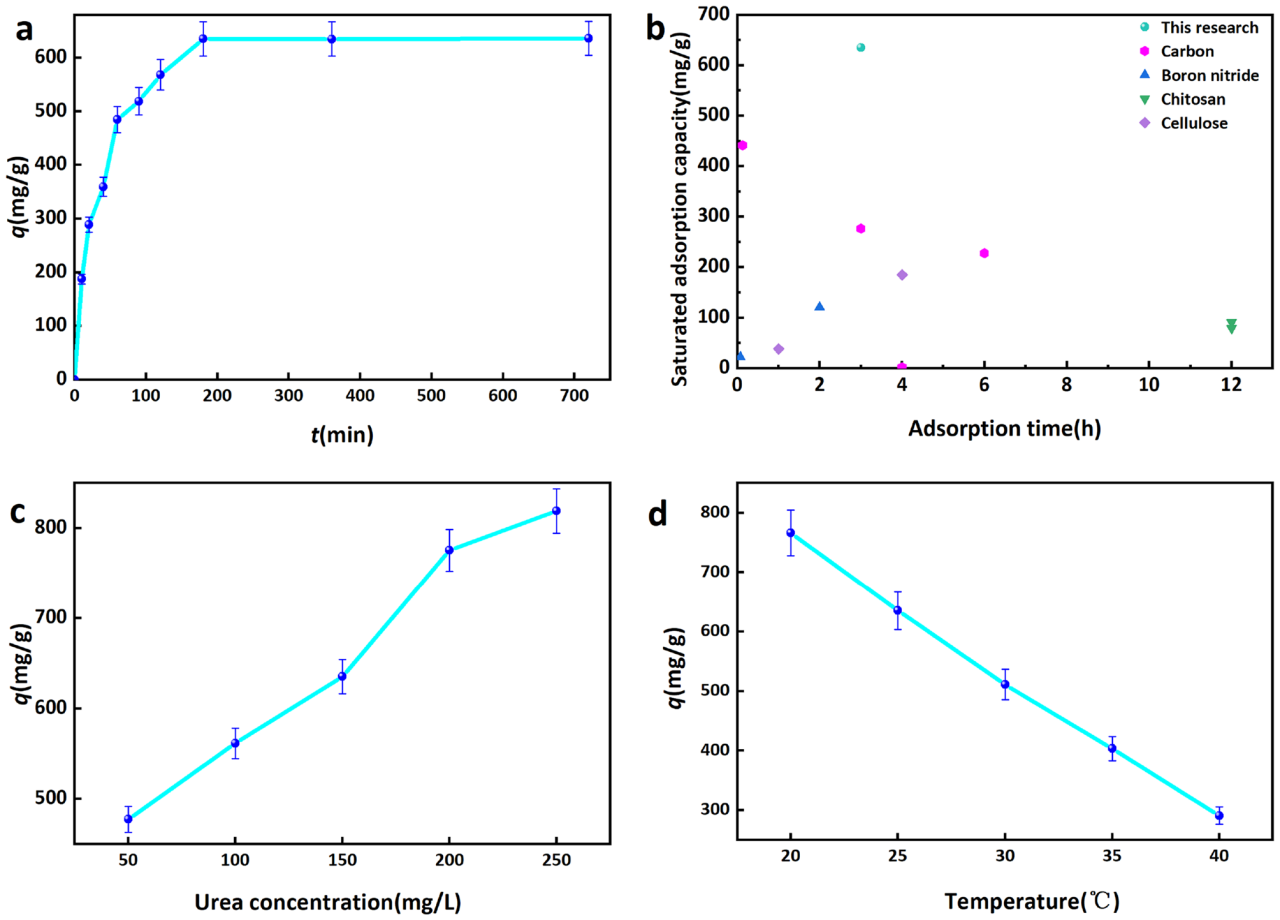
Effect of (**a**) adsorption time and (**b**) adsorption capacity comparison, (**c**) urea initial concentration (**d**) temperature on the adsorption capacity of urea.

We have considered the effect of treatment times on urea adsorption performance using the previously optimized CPB, uptake was measured at a fixed adsorption temperature of 25 °C, initial urea concentration of 150 mg/L of urea and dosage of 50 mg of CPB. The findings depicted in Fig. 8a indicate a positive correlation between the duration of time and the adsorption of urea. The urea adsorption rate by CPB in the early stage was high, with a discernible lower uptake rate after 1 h. This can be attributed to the combination of CPB pore structure and the presence of oxygen-containing functional groups, which have a high affinity for urea that is rapidly captured in the early stages. The presence of urea in the pores of CPB occupies the active sites of CPB, which reduces the active sites and leads to the saturation of intermolecular interactions, which reduces the uptake rate and ultimately saturates the adsorption. Adsorption reached equilibrium after 3 h with a maximal urea uptake of 635.26 mg/g. The adsorption capacity of CPB was found to be superior to that of conventional urea adsorbents, as depicted in Fig. 8b^8,51–59^.

#### Effect of initial urea concentration on the adsorption capacity of CPB

Experiments were conducted at a temperature of 25 °C, utilizing 50 mg CPB at adsorption saturation, to evaluate the impact of initial urea concentration on the adsorption performance. Fig. 8c illustrates the positive correlation between urea concentration and the adsorption capacity of CPB, as well as the concurrent increase in the rate of adsorption. The increase in urea concentration from 50 to 250 mg/L resulted in a corresponding increase in the adsorption capacity of CPB from 477.45 to 818.98 mg/g. An elevated urea concentration was found to enhance the likelihood of urea and CPB coming into contact, thereby expediting the infiltration of urea into the CPB pores and promoting interactions with amino and oxygen-containing functional groups. Consequently, this led to an augmentation in the adsorption capacity of the CPB, a phenomenon that was further corroborated by subsequent analysis of the adsorption kinetics.

#### Effect of temperature on the adsorption capacity of CPB

Experiments were conducted at a fixed CPB mass of 50 mg and urea concentration of 150 mg/L, operated at adsorption saturation in order to investigate the effect of temperature on performance. As shown in Fig. 8d, the amount of urea adsorbed decreased as the temperature was increased, with a maximum adsorption capacity of 766.11 mg/g at 20 °C. This indicates that temperature has a negative effect on adsorption, suggesting that urea uptake is an exothermic process favored by lower temperatures.

#### Effect of pH on the adsorption capacity of CPB

**Figure 9:**
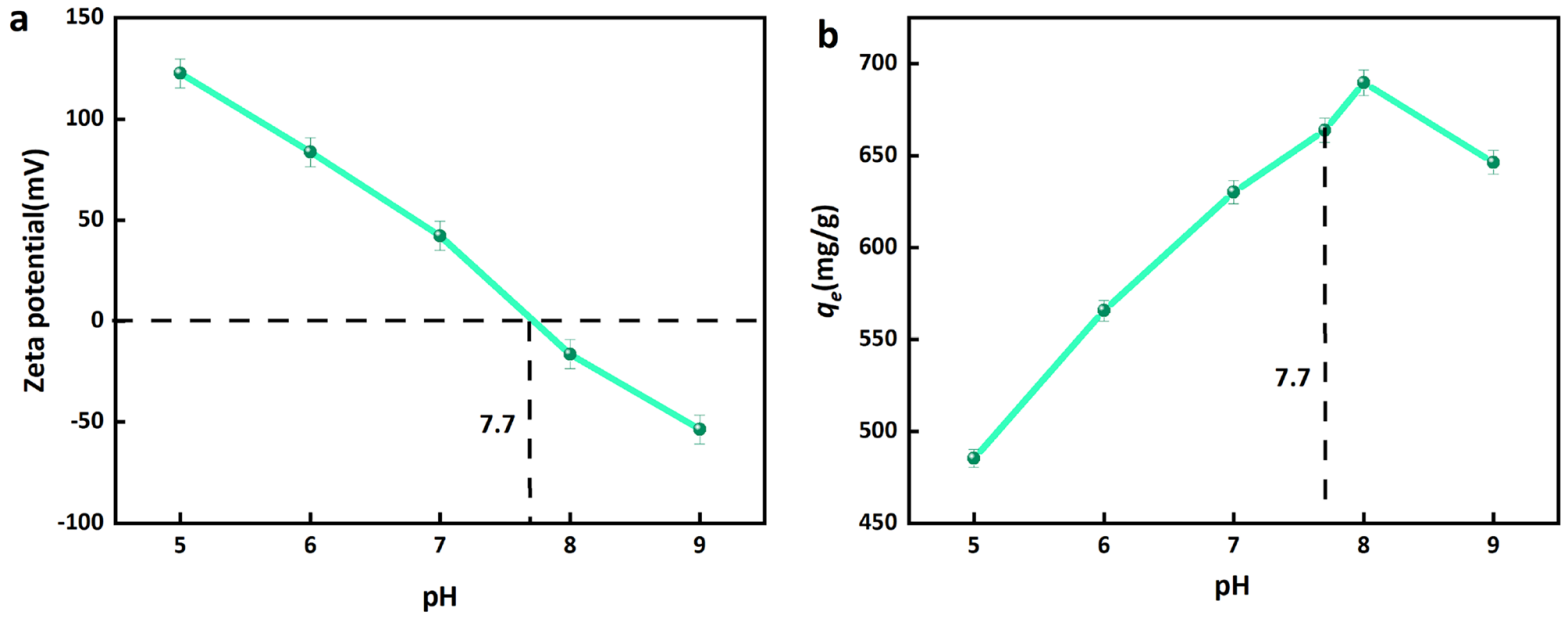
(**a**) Effect of pH on the adsorption capacity of urea, (**b**) The Zeta potentials of CPB under different initial pH.

The modification of the pH value of the solution has an influence on both the surface characteristics of CPB and the properties of urea. Consequently, an investigation was conducted to assess the impact of pH on urea adsorption performance at a temperature of 25 °C and a concentration of 150 mg/L. Fig. 9 presents the zeta potential and adsorption quantity of urea at various pH levels. At the outset, the adsorption capacity displayed a positive association with pH, peaking at 689.75 mg/g when pH equaled 8, subsequently declining as pH increased. Meanwhile, the zeta potential exhibits a decline as the pH level rises, attaining the isoelectric point at a pH value of 7.7, as visually depicted in Fig. 9a,b. In the presence of acidic conditions, a process of protonation takes place in the solution, as depicted in Eq. 6. The addition of H^+^ facilitates dissociation and induces a displacement of the equilibrium towards the right. The zeta potential analysis indicates that the charged particles (CPB) exhibit a positive charge when the pH is below 7.7, resulting in a reduction in adsorption due to electrostatic repulsion between entities possessing similar charges. In this case, an elevation in pH levels results in an increase in the quantity of adsorption^35,59^. When the pH level falls within the range of 7.7–8, the CPB charged state transitions to a negative state, resulting in a weakened electrostatic repulsive force. Consequently, this leads to an increase in the adsorption amount and adsorption force. With the continued introduction of OH^−^ ions, the presence of an alkaline NH_2_ group in urea results in electrostatic repulsion when in contact with the negative charge of CPB, thereby hindering the adsorption of urea^60–62^. Consequently, adsorption decreases with increasing pH. Therefore, the optimum pH for adsorption is 8.6

## Adsorption in actual wastewater

**Figure 10:**
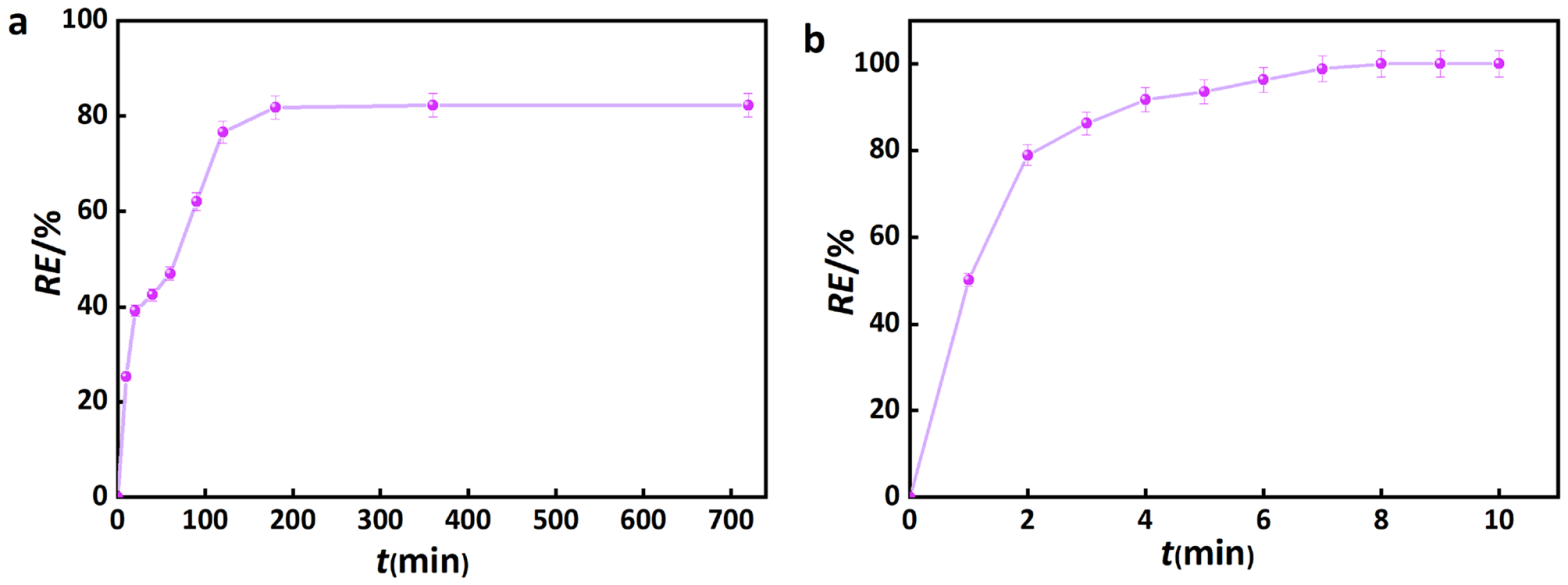
Curves of adsorption efficiency versus adsorption time for (**a**) domestic wastewater, (**b**) human urine.

Figure 10 illustrates the correlation between the adsorption efficiency and adsorption time of CPB in a realistic setting. In the context of domestic wastewater, the adsorption equilibrium can be achieved within 200 min, resulting in an 83% removal rate. Conversely, the human body can attain adsorption equilibrium within a mere 8 min, leading to a complete removal rate of 100%.

### Adsorption isotherm

This study utilized three adsorption isotherm models to simulate the process of adsorption. The Langmuir model^63^, which assumes adsorption takes place on homogeneous adsorbent sites, has demonstrated its efficacy in numerous practical adsorption processes. The Langmuir isotherm is frequently utilized in the context of chemical adsorption, wherein it is founded upon the occurrence of a one-to-one monolayer adsorption between the adsorption site and the adsorbed molecule. The expression of the Langmuir isotherm is as follows,

7 $$ q_{e} = \frac{{QbC_{e} }}{{1 + bC_{e} }} $$

Eq. 7 converted into the linear form as presented in Eq. 8,

8 $$ \frac{{C_{e} }}{{q_{e} }} = \frac{1}{{Q_{b} }} + \frac{{C_{e} }}{Q} $$

where *Q* (mg/g) denotes the maximum monolayer adsorption capacity, *C*_*e*_ signifies the equilibrium concentration of the adsorbate in solution, and *b* (mg/L) represents the Langmuir constant. The Freundlich isotherm model^64^ is often used for liquid phase adsorption, and is an empirical formulation that assumes adsorption on non-uniform surfaces, described by the following equation,

9 $$ \ln q_{e} = \ln Q_{f} + \frac{1}{n}\ln C_{e} $$

where *Q*_*f*_ represents the Freundlich isotherm constant, while *1/n* serves as an indicator of adsorption strength and determines the extent to which adsorbate uptake adheres to this particular isotherm model.

The Harkins–Jura^8^, formula is utilized to describe the phenomenon of multilayer adsorption occurring on surfaces that possess pores of varying sizes^8^. This formula is mathematically expressed by Eq. 10,

10 $$ \frac{1}{{q_{e}^{2} }} = \left( {\frac{{\text{B}}}{{\text{A}}}} \right) - \left( {\frac{1}{{\text{A}}}} \right){\text{log}}C_{e} $$

where A and B are variable constants of the Harkins–Jura equation, A and B are determined from the slope and intercept of the linear fit plot (Table 5).

**Figure 11:**
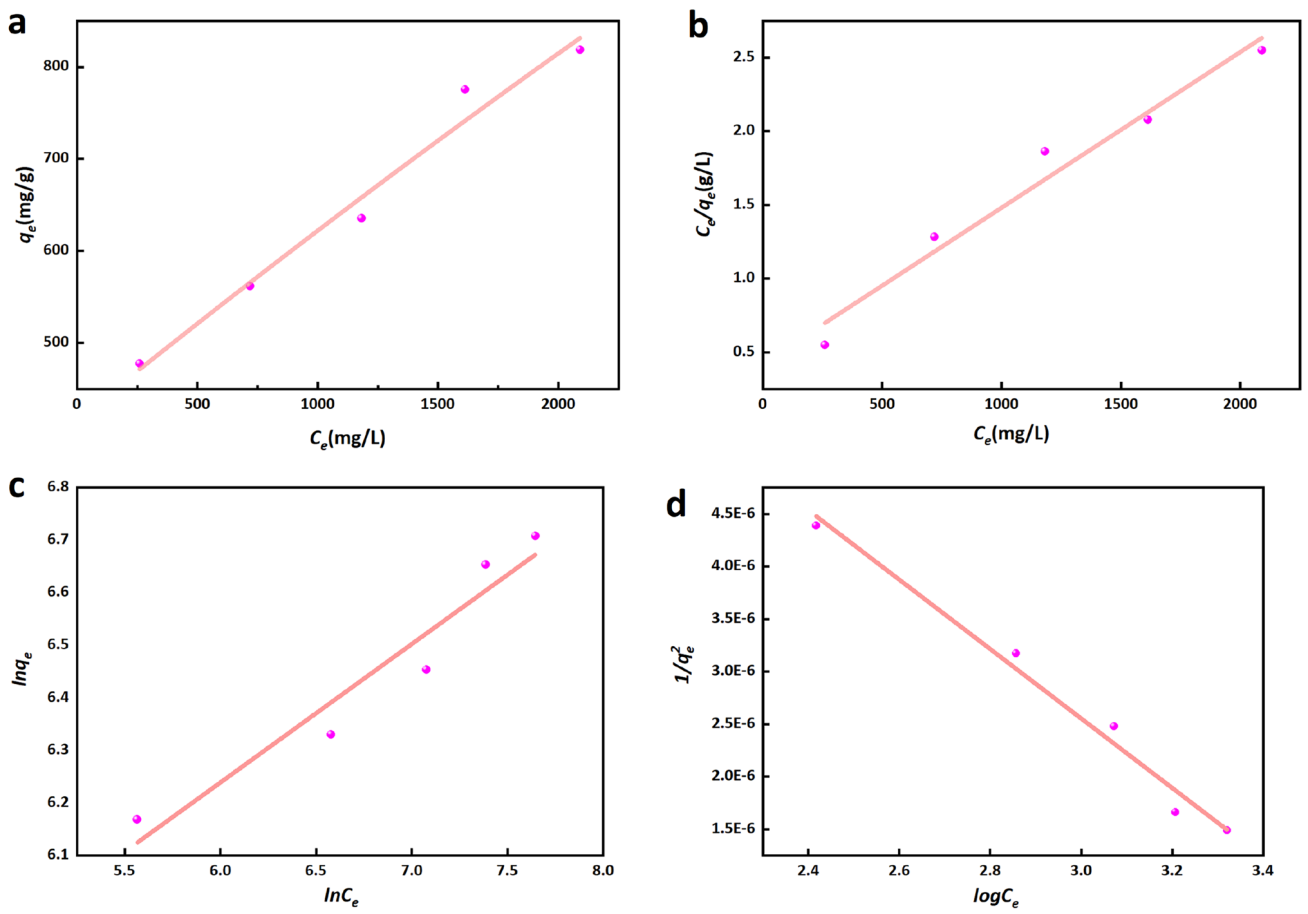
(**a**) Adsorption isotherm of urea adsorbed on CPB, (**b**) Langmuir plot, (**c**) Freundlich plot, (**d**) Harkins–Jura plot.

Based on the Eq. 3, the isotherm for urea adsorption on CPB can be determined by examining the correlation between *q*_*e*_ and *C*_*e*_, as illustrated in Fig. 11a. It is evident that an increase in *C*_*e*_ leads to a corresponding increase in *q*_*e*_. Table 5 presents the equilibrium isotherm constants and correlation coefficients obtained through linear regression analysis of the Langmuir, Freundlich, and Harkins–Jura isotherms. The linear forms of the Langmuir, Freundlich, and Harkins–Jura plots are shown in Fig. 11b–d.

**Table 5:** List of constants calculated from different models.

Langmuir			Freundlich			Harkins–Jura		
*Q* (mg/g)	*b* (mg/L)	*R*^2^	*Q*_*f*_ (mg/g)	*n*	*R*^2^	*A*	*B*	*R*^2^
2.36	399.54	0.96	105.58	3.80	0.93	302,913.70	3.77	0.98

A linear relationship between *C*_*e*_*/q*_*e*_ and *C*_*e*_ is obtained using Eq. 7, as shown in Fig. 11b. The parameters *Q* and *b* are derived from the slope and intercept, representing the urea adsorption capacity of CPB. Using different dosage of CPB, the relationship in terms of urea concentration exhibited a good fit to the Langmuir model; correlation coefficient *R*^2^ = 0.96. As noted in Table 5, urea uptake on CPB generated a calculated maximum monolayer capacity *Q* of 2.36 mg/g. Application of the Freundlich isotherm generated n greater than 1, suggesting a favorable adsorption^65^. However, the Freundlich isotherm exhibits a lower correlation coefficient (*R*^2^ = 0.93), suggesting a weaker concordance between the Freundlich model and the empirical data. In the case of the Harkins–Jura formula, the *R*^2^ = 0.98 indicates a much better correlation, suggesting that urea adsorption follows the Harkins–Jura model.

### Kinetic model of urea adsorption on CPB

The urea adsorption kinetics on CPB was investigated using pseudo-first-order and pseudo-second-order models. First-order kinetics can be assessed using the rate expression of Lagergren^59^, given is Eq. 11,

11 $$ \log \left( {q_{e} - q} \right) = \log q_{e} - \frac{{k_{1} t}}{2.303} $$

where *q*_*e*_ and *q* (mg/g) denote the quantities of urea adsorbed on CPB at equilibrium and time *t*, respectively, while *k*_1_ (min^−1^) represents the first-order rate constant^59,66^.

The second-order kinetic model^1^ can be expressed using Eq. 12,

12 $$ \frac{t}{q} = \frac{1}{{k_{2} q_{e}^{2} }} + \frac{t}{{q_{e} }} $$

where the second-order adsorption rate constant, denoted as *k*_*2*_ (g·min/mg), is responsible for the observed linear relationship between *t/q* and *t*^59,66^.

**Figure 12:**
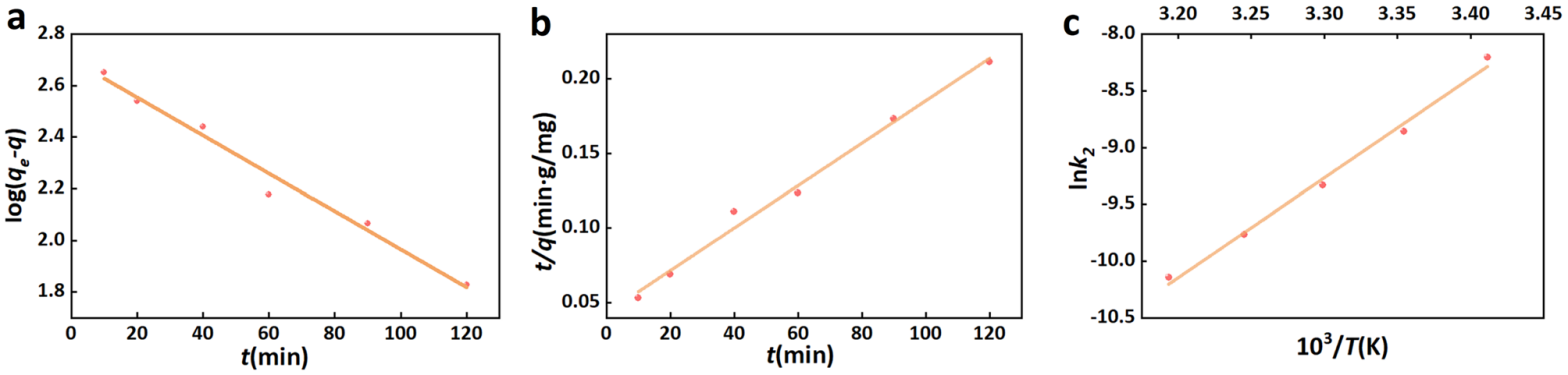
(**a**) Mapping of pseudo first-order models, (**b**) Mapping of pseudo second-order models, (**c**) Mapping of lnk_2_ and reciprocal temperature.

The results of the kinetic model fitting (including correlation coefficient, *R*^2^) are presented in Table 6, and the associated plots are given in Fig. 12a,b. The first-order model delivered a low correlation coefficient (*R*^2^ = 0.97), and the calculated *q*_*e*_ deviated significantly from the experimental *q*_*e*_ values.

**Table 6:** The first-order and second-order adsorption rate constants.

*q*_*e,exp*_ (mg/g)	First-order kinetic model			Second-order kinetic model		
	*k*_1_ (min^−1^)	*q*_*e,cal*_ (mg/g)	*R*^2^	*k*_2_ (g min/mg)	*q*_*e,cal*_ (mg/g)	*R*^2^
635.26	1.69 × 10^−2^	504.78	0.97	4.66 × 10^−5^	704.23	0.99

The *t/q* versus* t* plots, depicted in Fig. 12b, are utilized for the determination of the second-order rate constants *k*_*2*_ and *q*_*e*_. It is evident from the graph that the empirical data aligns favorably with the second-order kinetic model. The calculated model parameters are included in Table 6 with the correlation coefficient that exceeded 0.99. The findings of this study suggest that the adsorption of urea on CPB follows a second-order dynamic model. The activation energy for urea adsorption was determined through the utilization of the Arrhenius equation,

13 $$ \ln k_{2} = \ln k_{0} - \frac{{E_{a} }}{RT} $$

The value of *k*_2_ was determined at various temperatures ranging from 20 to 35 °C. In this context, *R* represents the gas constant with a value of 8.314 J/K·mol. Additionally, *k*_0_ (g·min/mg) denotes a factor that remains unaffected by changes in reaction temperature. *E*_*a*_ (kJ/mol) signifies the apparent activation energy of adsorption, while *T* (K) represents the thermodynamic temperature of the solution. The linear relationship resulting from a plot of ln*k*_*2*_ versus *10*^3^*/T* calculated according to Eq. 12 is shown in Fig. 12c. The correlation coefficient (*R*^2^) is 0.99, and the activation energy calculated from the slope is − 73.22 kJ/mol. A negative activation energy indicates a higher likelihood of urea adsorption and explains the decline in adsorption capacity as temperature rises.

### Mechanism of urea adsorption on CPB

**Figure 13:**
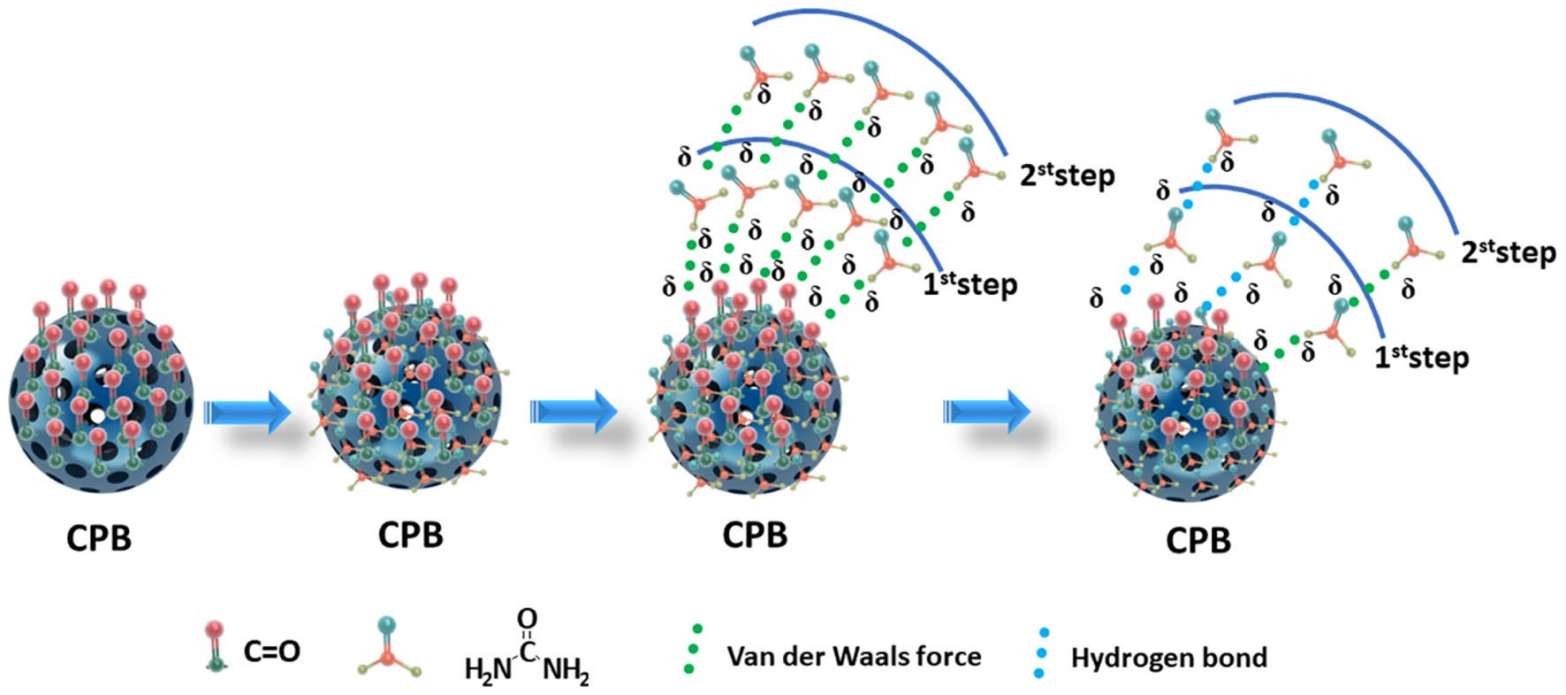
Sketch of the adsorption mechanism.

It has been reported that urea uptake on carbon materials generally involves physical adsorption. Nevertheless, the forces implicated in physical adsorption exhibit a low magnitude, characterized by activation energies below 4.2 kJ/mol. Kameda et al. have reported the activation energy for urea separation by adsorption on activated carbon spheres was − 71.6 kJ/mol, and classified the process as physical adsorption^8^. The activation energy for urea adsorption on CPB was − 73.22 kJ/mol. Therefore, we propose that urea adsorption on CPB follows physical adsorption. The adsorption mechanism is illustrated in Fig. 13.

In the present system, physisorption is characterized by the presence of van der Waals forces, electrostatic force and hydrogen bonding, with hydrogen bonding being the prevailing interaction. The van der Waals force is known to be active when organic molecules are adsorbed from aqueous solutions^8^. The zeta potential test results presented support for the existence of electrostatic interactions between CPB and the urea solution. The adsorption is reduced in the case where CPB and the urea solution possess identical charges due to the influence of electrostatic forces. The formation of hydrogen bonds between the C=O on the surface of CPB and the NH_2_ of urea is regarded as a crucial element in the process of adsorption. Furthermore, the interaction between another NH_2_ from a second urea molecule and the C=O of the urea molecule already adsorbed on CPB leads to the formation of supplementary hydrogen bonds^13^. Based on the FT–IR and XPS analysis, dehydration involving the amino group of the urea molecule and the carboxyl group on CPB results in chemisorption.

**Figure 14:**
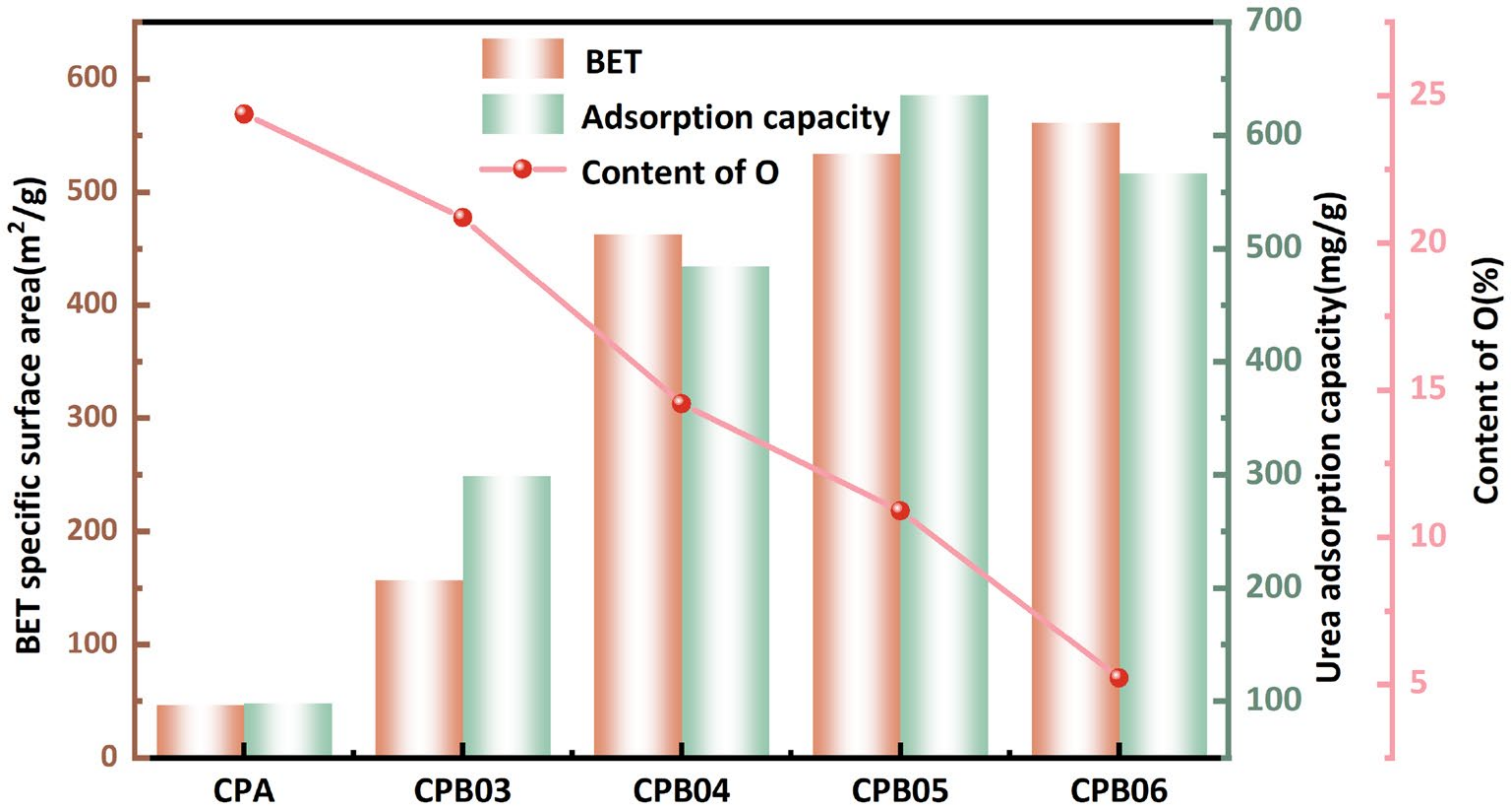
Plot of adsorption capacity, specific surface area and oxygen content.

Simultaneously, the rich pore structure inherent in CPB significantly influences its properties, with the specific surface area expanding as the CPB preparation temperature rises, albeit at the expense of decreased oxygen content. In order to delve deeper into the impact of pore size and oxygenated functional groups on adsorption, a comparison was made of the adsorption capacities of CPBs and CPA prepared at varying temperatures, as illustrated in Fig. 14. The results indicate a trend of increasing adsorption capacity followed by a decrease as the preparation temperature rises. The highest adsorption capacity was observed for the rise in specific surface area from CPB05 to CPB06 is 27.5 cm^−2^/g, yet the adsorption capacity diminishes concurrently, indicating a heightened significance of oxygen-containing functional groups. However, it is important to note that the oxygen content of CPB06 is only 5.21%, resulting in a decrease in adsorption amount as the specific surface area increases. Hence, it can be inferred that as temperature rises, pore size significantly influences adsorption initially, but as temperature surpasses a certain threshold, the increase in specific surface area diminishes, resulting in a weakening of the impact of pore size on CPB adsorption and a greater emphasis on the role of oxygen-containing functional groups.

Considering all instances collectively, it can be inferred that the adsorption of CPB onto urea is a mechanism of adsorption influenced by both physisorption and chemisorption. These interactions lead to multilayer adsorption and are supported by the Harkins–Jura and pseudo-second order reactions models.

## Conclusion

In this study, a low cost catalytic hydrothermal process was used to produce porous biochar with high yield from agricultural waste corncobs. The biochar demonstrated a high adsorption capacity of urea (635.26 mg/g) under optimum conditions (500 °C, 6 h and 15 °C/min). The remarkable capacity of CPB for adsorbing urea can be ascribed to a high surface concentration of oxygen-containing functional groups in conjunction with its extensive specific surface area. Urea uptake by CPB is a spontaneous exothermic multilayer adsorption involving physical and chemical adsorption. The process is well described by the Harkins–Jura and the pseudo-second order kinetic models. The results presented in this study provide a theoretical basis for the scale-up of adsorbent production and practical application as adsorbent. The CPB exhibits potential as a bio-derived adsorbent for the retrieval and elimination of urea from nitrogenous wastewater and hemodialysis. Our work can provide a new direction for the beneficial use of agricultural waste that alleviates the associated environmental burden.

## Data Availability

The datasets generated and/or analysed during the current study are not publicly available due [REASON WHY DATA ARE NOT PUBLIC] but are available from the corresponding author on reasonable request.
